# Small Extracellular Vesicles from Peripheral Blood of Aged Mice Pass the Blood-Brain Barrier and Induce Glial Cell Activation

**DOI:** 10.3390/cells11040625

**Published:** 2022-02-11

**Authors:** Diana M. Morales-Prieto, José M. Murrieta-Coxca, Milan Stojiljkovic, Celia Diezel, Priska E. Streicher, Julian A. Henao-Restrepo, Franziska Röstel, Julia Lindner, Otto W. Witte, Sebastian Weis, Christian Schmeer, Manja Marz

**Affiliations:** 1 Placenta Lab, Department of Obstetrics, Jena University Hospital, 07747 Jena, Germany; josemartin.murrietaCoxca@med.uni-jena.de (J.M.M.-C.); priska.streicher@med.uni-jena.de (P.E.S.); julian.andres.henao.restrepo@uni-jena.de (J.A.H.-R.); 2 RNA Bioinformatics and High Throughput Analysis, Friedrich Schiller University Jena, 07743 Jena, Germany; celia.diezel@leibniz-ipht.de; 3 Hans-Berger Department of Neurology, Jena University Hospital, 07747 Jena, Germany; milan.stojiljkovic@med.uni-jena.de (M.S.); julia.lindner@med.uni-jena.de (J.L.); otto.witte@med.uni-jena.de (O.W.W.); christian.schmeer@med.uni-jena.de (C.S.); 4 FLI Leibniz Institute for Age Research, 07745 Jena, Germany; 5 Department for Anesthesiology and Intensive Care Medicine, Jena University Hospital, 07747 Jena, Germany; franziska.roestel@med.uni-jena.de; 6 Leibniz Institute for Natural Product Research and Infection Biology, Hans Knöll Institute, 07745 Jena, Germany; sebastian.weis@med.uni-jena.de; 7 Institute for Infectious Disease and Infection Control, Jena University Hospital, 07747 Jena, Germany

**Keywords:** extracellular vesicles, exosomes, sEV, blood-brain barrier, glia, neuroinflammation

## Abstract

Extracellular vesicles (EVs), including small EVs (sEVs), are involved in neuroinflammation and neurodegenerative diseases, including Alzheimer’s disease, Parkinson’s disease, and amyotrophic lateral sclerosis. Yet, increased neuroinflammation can also be detected in the aging brain, and it is associated with increased glial activation. Changes in EV concentration are reported in aging tissues and senescence cells, suggesting a role of EVs in the process of aging. Here, we investigated the effect of peripheral sEVs from aged animals on neuroinflammation, specifically on glial activation. sEVs were isolated from the peripheral blood of young (3 months) and aged (24 months) C57BL/6J wildtype mice and injected into the peripheral blood from young animals via vein tail injections. The localization of EVs and the expression of selected genes involved in glial cell activation, including *Gfap*, *Tgf-β*, *Cd68*, and *Iba1*, were assessed in brain tissue 30 min, 4 h, and 24 h after injection. We found that sEVs from peripheral blood of aged mice but not from young mice altered gene expression in the brains of young animals. In particular, the expression of the specific astrocyte marker, *Gfap*, was significantly increased, indicating a strong response of this glial cell type. Our study shows that sEVs from aged mice can pass the blood-brain barrier (BBB) and induce glial cell activation.

## 1. Introduction

Extracellular vesicles (EVs) constitute a heterogeneous group of specialized membranous vesicles involved in intercellular communication [1]. Small EVs (sEVs), also known as exosomes, are formed intracellularly via endocytic invagination and are generated by outward budding at the endosomal membrane of the multivesicular bodies (MVBs) [2]. Experimental evidence was provided recently suggesting that circulating sEVs may act as neuroinflammatory mediators in systemic inflammation [3].

Neuroinflammation plays a critical role in brain aging [4,5], and it is a common pathological feature of neurodegenerative diseases, including Alzheimer’s disease (AD), Parkinson’s disease (PD), frontotemporal lobar dementia (FTD), and amyotrophic lateral sclerosis (ALS). Consistently, neuroinflammation is characterized by glial activation and pro-inflammatory cytokine production by the central nervous system (CNS) resident cells [6].

Accumulating evidence implicates EVs in the aging process, e.g., plasma EV concentration decreases with age in humans. In this context, EVs from the elderly are preferentially taken up by B cells and monocytes [7]. Plasma EVs isolated from young donors but not from elderly donors promote the osteogenic differentiation of mesenchymal stem cells in a galectin-3-dependent manner [8]. Furthermore, EVs purified from the elderly suppress cell proliferation and osteogenic differentiation of bone marrow stromal cells [9].

More recently, sEVs secreted by stem/progenitor cells of the healthy hypothalamus were associated with a slowing of aging [10]. In particular, the process of aging was suggested to be controlled by exosomal miRNAs from the hypothalamic stem cells, further supporting the role of EVs in the aging process.

Brain aging and age-associated neurodegenerative diseases, such as Alzheimer’s disease, are characterized by an increase in cellular levels of inflammatory mediators, including TNF-α, IL-6, TGF-β, and gliosis induced by glial cell activation [11]. The introduction of blood from young mice into old mice increased cognitive abilities and synaptic connectivity, suggesting that young blood contains specific factors supporting cognitive function. On the contrary, injection of old blood led to cognitive decline [12].

The impact of peripheral blood-derived sEVs on aging, in particular in the CNS, has not been assessed yet. Thus, putative targets of peripheral sEVs, in particular in the aging brain, remain to be elucidated. Here, we evaluated uptake of peripheral blood sEVs from aged and young wild-type mice and the transcriptional changes in vivo and in vitro. We focused on the sEV ability to pass the BBB and to impact neuroinflammation and glial cell activity.

## 2. Materials and Methods

### 2.1. Animal Experiments

C57BL/6J mice were bred and maintained at the animal facility (ZET) of the University Hospital Jena under specific pathogen-free conditions with a 14 h light/10 h night cycle and fed *ad libitum*. For sEV isolation, blood from old (24 months) or young (<3 months) animals was collected in citrate collection tubes (*n* = 5). Recipient animals (11–12-week-old mice) were injected with 2 µg/100 µL of protein equivalent of sEVs or saline solution as the vehicle into the tail vein. Mice were sacrificed 0.5 h, 4 h, and 24 h after injection (*n* = 3). Blood was collected in heparinized syringes via cardiac puncture and mice were subsequently perfused with PBS. Brains were harvested for immunostaining and RNA analysis, as described below.

For in vitro assays, a mixed culture of microglia and astrocytes from newborn animals (*n* = 3) was performed following the protocol developed in our lab [13,14]. Briefly, whole brains were depleted from meninges and then enzymatically digested. The resulting cell suspension was centrifuged onto a gradient density separation using Percoll (GE Healthcare, Freiburg, Germany, #17-5445-02). Pellets were washed and glial cells were placed in T75 cell culture flasks coated with poly-L-lysine and incubated at 37 °C and 5% CO_2_ for 14 days.

### 2.2. sEV Isolation and Labeling

sEVs were isolated from pooled plasma (approximately 480 µL/mouse; five mice per condition) by differential ultracentrifugation. Samples were centrifuged at 10,000× *g* for 10 min and then at 18,900× *g* for 30 min at 4 °C to remove cell debris and large vesicles. Supernatants were filtrated through a 0.22 µm membrane filter and then centrifuged twice at 100,000× *g* for 70 min at 4 °C using a Beckman Coulter Type 55Ti rotor (Beckman Coulter, Krefeld, Germany). sEVs were labeled with 2 µM PKH67 dye for 5 min (Sigma-Aldrich, Munich, Germany). The reaction was stopped by adding an equal volume of 1% BSA/NaCl 0.9%. Labeled sEVs were washed twice with saline solution by centrifugation at 100,000× *g* for 70 min. Pellet was resuspended in 100 µL saline solution and protein was quantified by Micro BCA^TM^Protein assay (ThermoFisher Scientific GmbH, Dreieich, Germany). Naive recipient mice received 2 µg/100 µL of protein equivalents of sEV_PKH-67_. This amount was assumed to distribute into 3 mL of blood and produce a blood level of 0.6 µg/mL, which represents about 1/75th the concentration of sEVs found in the mouse blood [15]. The injected amount of exosome protein represents about 2.6 × 10^12^ sEVs, or 9.0 × 10^11^ sEV particles per mL of blood [15].

### 2.3. sEV Characterization by Nanoparticle-Tracking Analysis (NTA), Western Blot Analysis, and Cryo-Transmission Electron Microscopy (Cryo-TEM)

Size and concentration of EV suspensions were assessed by NTA on a NanoSight version NS500 (NanoSight Ltd., Amesbury, UK), as described before [16,17]. Protein equivalents of sEV fractions were also quantified using Micro BCA assay (Pierce™ BCA Protein Assay Kit, Sigma-Aldrich, Poole, UK) under the manufacturer’s instructions. Briefly, 150 μL of the sample were incubated with 150 μL of the working reagent for 2 h at 37 °C. Absorbance was then measured at 562 nm using a SPECTROstar microplate reader. sEV concentrations were determined based on a standard curve of BSA and used as protein equivalents for further experiments. For the analysis of EV markers, sEV fractions (5 µg) were mixed with non-reducing Laemmli-loading buffer (375 mM Tris.HCl, 9% SDS, 50% glycerol, 0.03% bromophenol blue), loaded on a 12% precast gel SERVAGel™ (SERVA Electrophoresis GmbH, Heidelberg, Germany), and resolved proteins were transferred to a nitrocellulose membrane (Hybond-P; GE Healthcare, Freiburg, Germany). Non-specific binding sites were blocked by incubation with TBST containing 5% (*w*/*v*) non-fat dried milk for 1 h at room temperature. Membranes were immunoblotted with specific primary antibodies overnight at 4 °C, followed by 1 h incubation at room temperature with the respective HRP-conjugated secondary antibody.

The following primary antibodies were used at a 1:500 dilution: rabbit anti-mouse Alix (Cell Signaling Technology, Frankfurt am Main, Germany, Cat-Nr. 92880); mouse anti-mouse/human CD63 (Invitrogen, ThermoFisher Scientific GmbH, Dreieich, Germany, Cat-Nr. 10628D); rabbit anti-mouse TSG101 (Invitrogen, ThermoFisher Scientific GmbH, Dreieich, Germany Cat-Nr. PA5-82236); and rat anti-mouse CD31 (Abcam, Berlin, Germany, Cat-Nr. ab56299). For detection, the respective goat anti-rat HRP (Cell Signaling Technology, Frankfurt am Main, Germany Cat-Nr. 7077S) or anti-rabbit HRP (Cell Signaling Technology, Frankfurt am Main, Germany Cat-Nr. 7074P2) secondary antibodies were diluted 1:3000. Blots were developed using an enhanced chemiluminescence (ECL) detection kit (Millipore, Schwalbach, Germany). The intensity of bands was analyzed by a MF-ChemiBis 3.2 gel documentation system with Totallab TL100 software version 2006 (Biostep GmbH, Jahnsdorf, Germany).

For Cryo-TEM, a small droplet (5 μL) of the isolated sEV sample was placed on a TEM grid Quantifoil UltrAuFoil holey gold film (R 1.2/1.3, 400 mesh; Quantifoil Micro Tools GmbH, Großlöbichau, Germany). Excess liquid was blotted for about one second between two strips of filter paper. Subsequently, the sample was rapidly plunge-frozen in liquid ethane (cooled to about −180 °C by liquid nitrogen) in a Zeiss Cryobox (Carl Zeiss GmbH, Oberkochen, Germany). The frozen specimen was transferred into a precooled CM120 cryo-transmission electron microscope (Philips, Eindhoven, The Netherlands) using a Gatan 626-DH cryo-holder (Gatan, Pleasanton, CA, USA) The Cryo-TEM was operated at 120 kV, and the samples were imaged under low-dose conditions. The images were recorded with a 2K CMOS Camera (F216 and EMMENU V4.0 software; camera and software TVIPS GmbH, Munich, Germany).

### 2.4. Assessment of sEV Uptake by Immunofluorescence

To localize peripheral sEVs in the in vivo model, brain tissue was harvested and cryopreserved in sucrose. Tissues were embedded in optimal cutting temperature compound and sectioned on a Leica CM1850 cryostat at a thickness of 40 µm. Sections were attached to microscope slides and allowed to dry on a slide warmer for 1 h at 37 °C. Tissues were permeabilized and blocked with 0.3% Triton X- 100/3% NDS (normal donkey serum) in PBS for 30 min, washing the slides with PBS between the steps. The primary antibody rat anti-CD31 (Abcam, Berlin, Germany, Cat-Nr. ab56299) diluted 1:250 was used. Slides were incubated in a dilution of 1:500 in PBS/10% BSA overnight at 4 °C. After washing with PBS, secondary rhodamine antibodies (Jackson Immunoresearch, West Grove, PA, USA) and DAPI (4-6-Diamidino-2-phenylindole, Sigma-Aldrich, Munich, Germany) were added in a dilution of 1:500 in PBS/10% BSA and incubated for 1 h at room temperature. Images of coronal brain slices (Supplementary Figure S1) were acquired using automatic tiling and stitching tools of the Axio Observer microscope (Carl Zeiss, Oberkochen, Germany). High magnification images were obtained in the cortex in slices of the medial brain, in approximately bregma −2.0 mm.

For the in vitro assays, primary glial cultures containing 80% astrocytes and 20% microglia [13] were grown in an 8-chamber culture slide (Falcon™, ThermoFisher Scientific GmbH, Dreieich, Germany, Cat no. 10364551) of 10,000 cells per well and, after washing with PBS, incubated with 2 µg of PKH67-labeled sEVs for 24 h in DMEM containing exosome-depleted (ED)-FBS. For immunodetection, cells were fixed with 4% PFA (paraformaldehyde) for 30 min at room temperature (RT). Slides were blocked with 0.1% BSA during 20 min at RT. Primary antibodies rabbit anti-CD11b (Abcam, Berlin Germany, Cat-Nr. ab128797), rabbit anti-Iba1 (WAKO Chemicals, Richmond, VA, USA, Cat no. 019-19741), and chicken anti-GFAP (Encor, Gainesville, FL, USA. Cat-Nr. ABIN 4956156) diluted 1:50 in PBS/1% BSA were applied. Cell slides were incubated 2 h at 37 °C in a humidified chamber. The secondary antibodies, anti-rabbit-AF647 (ThermoFisher Scientific GmbH, Dreieich, Germany, Cat-Nr. A21246) and anti-chicken-rhodamine (Dianova, Hamburg, Germany Cat-Nr. 703-295-155) diluted 1:200 in PBS/1% BSA were used. Slides were incubated 1 h at 37 °C in a humidified chamber. DAPI was used at 1 μg/mL for nuclei staining. Slides were mounted with VECTASHIELD (Vector Laboratories, Burlingame, CA, USACat no. H-1000-10) and observed under an Axio Observer Z.1 confocal microscope (Carl Zeiss, Jena, Germany).

### 2.5. Analysis of Gene Expression 

Brain tissues of peripheral-EV recipient mice were snap-frozen in liquid nitrogen and stored at −80 °C until use.

For in vitro analyses, mixed glial cultures were seeded at 100,000 cells per well and incubated with 2 μg/mL of sEV protein equivalent for 24 h. The total RNA from mixed glial cultures was extracted using TRIzol reagent (ThermoFisher Scientific, Waltham, MA, USA, Cat no. 15596026), according to the manufacturer’s instructions. The RNA concentration, quality, and integrity were determined using a Nanodrop (ThermoFisher Scientific, Waltham, MA, USA). mRNA levels were determined by reverse transcription using the High-Capacity RNA-to-cDNA™ Kit (Applied Biosystems Deutschland GmbH, Darmstadt, Germany, Cat no. 4388950). Gene expression was assessed in two different laboratories using either TaqMan assays or SYBR Green primers, as described in the figure legends. Quantitative real-time PCR was performed using TaqMan assays (*Gfap*, Assay ID: Mm01253033_m1; *Iba1*, Assay ID: Mm00479862_g1; *Cd11b*, Assay ID: Mm00434455_m1; *Cd68*, Assay ID: Mm00839636_g1; *Cdkn2 (p16)*, Mm00494449_m1; *Cdkn1a* (*p21)*, Assay ID: Mm00432448_m1; *Tgf-β*, Assay ID: Mm01178820_m1; *Nos2 (*iNOS*)*, Assay ID: Mm00440502_m1; *Il1-β*, Assay ID: Mm00434228_m1; *Il-6*, Assay ID: Mm00446190_m1; *Tnf-α*, Assay ID: Mm00443258_m1; and *Gapdh*, Assay ID: Mm99999915_g1) and TaqMan Universal PCR Master Mix reagents (Applied Biosystems Deutschland GmbH, Darmstadt, Germany, Cat no. 4440048). The SYBR Green primers listed in Table 1 were designed for SYBR Green qPCR Protocol. PCR was run on a Mx3005P qPCR System (Applied Biosystems Deutschland GmbH, Darmstadt, Germany). mRNA expression was normalized using the 2^−ΔΔCt^ method relative to *Gapdh* and the respective control.

### 2.6. Statistical Analysis

Experiments were repeated independently at least three times. An unpaired *t*-test with Welch’s correction and one-way ANOVA with Dunnett’s multiple comparison test were performed for comparisons using Prism software version 9 (GraphPad, San Diego, CA, USA). A *p*-value < 0.05 was considered significant.

## 3. Results

### 3.1. Isolation and Characterization of sEV Fractions from Young and Aged Mice

**Figure 1:**
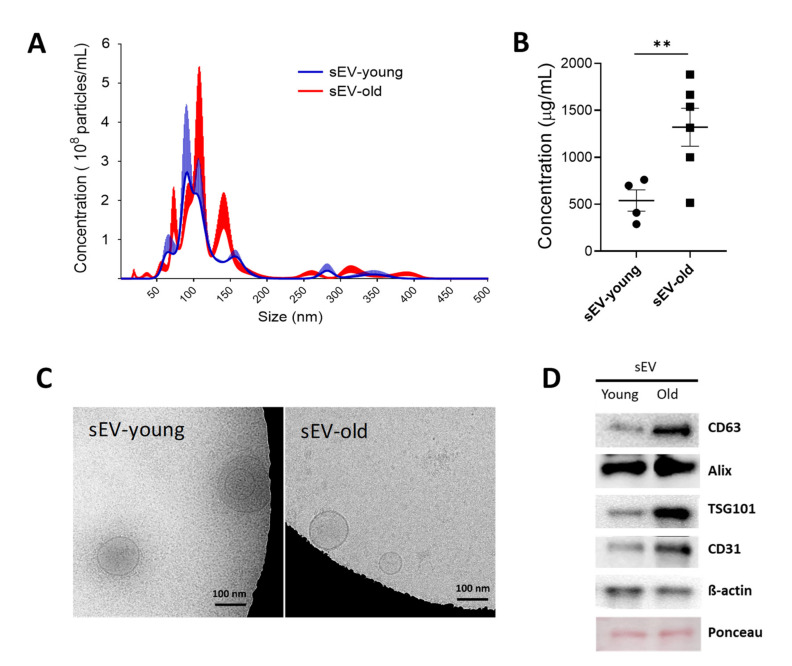
Characterization of sEVs isolated from plasma of young (3-month-old) and old mice (24-month-old). sEVs were enriched by differential ultracentrifugation. (**A**) Nanoparticle-tracking analysis (NTA) of sEV fractions from young (blue line) and old (red line) mice blood plasma. The graph shows sEV concentration and size, mean ± SEM (*n* = 3). (**B**) Protein equivalents measured by microBCA. (**C**) Cryo-TEM on purified sEVs. (**D**) Western blotting for sEV- and endothelial-associated markers. ** p < 0.01 from unpaired *t*-test with Welch’s correction.

sEV fractions were isolated from 4 mL plasma, obtained from 3- or 24-month-old mice (young and old mice, respectively), and quantified by NTA. Compared to young mice, a significant increase in amount (old: 3.25 × 10^8^ ± 2.15 × 10^8^ particles/mL vs. young: 2.32 × 10^8^ ± 9.83 × 10^7^ particles/mL) and size (old: 114.3 ± 13.5 nm vs. young: 97.4 ± 4.7 nm) of plasma sEVs was observed in old animals (Figure 1A). This was further confirmed by assessing EV protein equivalents via microBCA assay (old: 1319.72 ± 184.83 µg sEV/mL vs. young 539.56 ± 87.9 µg sEV/mL plasma) (Figure 1B). Cryo-TEM showed the presence of purified sEVs with round structures < 200 nm in diameter (Figure 1C). The expression of specific sEV markers (CD63, ALIX, and TSG101) and endothelial cell markers (CD31) was confirmed by western blotting (Figure 1D).

### 3.2. Localization of Peripherally Injected sEV in Recipient Mice

**Figure 2:**
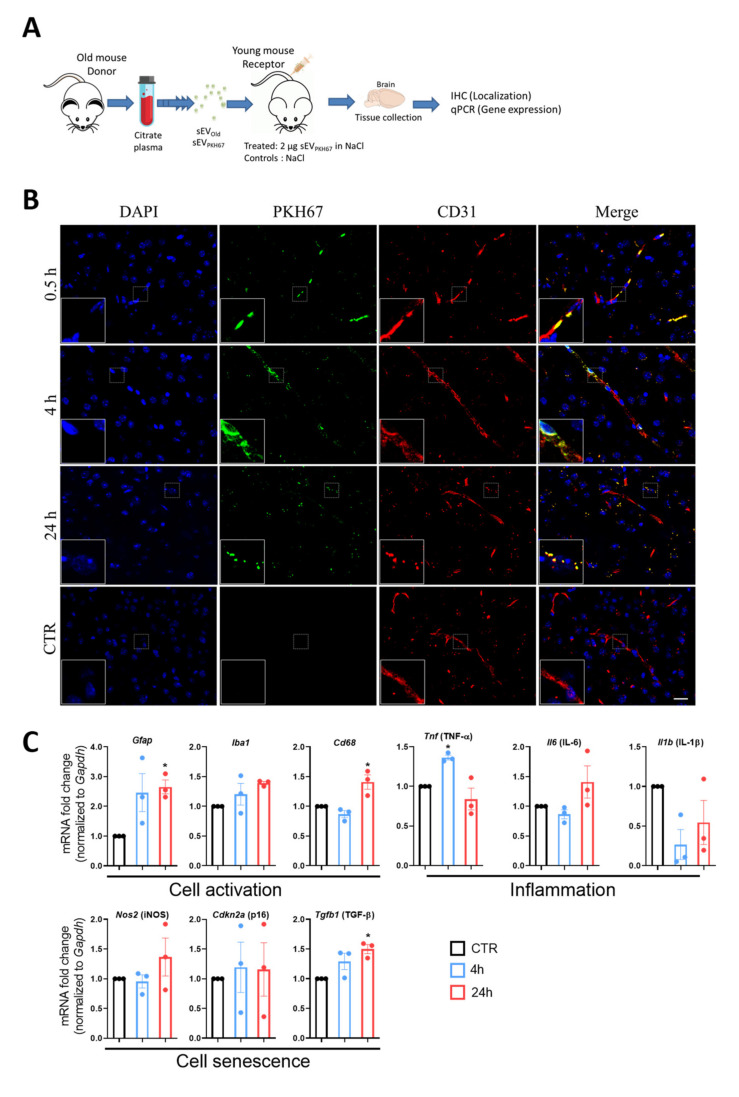
Localization and effects of peripherally injected sEVs in vivo. (**A**) Scheme of sEV treatment. sEVs were isolated from plasma of 24-month-old donor mice and stained with PHK67. Two µg-labeled sEVs were injected into the tail veins of 3-month-old mice. Brains were collected at 0.5, 4, and 24 h. (**B**) The representative confocal microscopic images show exogenous-labeled sEVs localizing mostly in the vascular compartment of the brain (at 30 min) and in the parenchyma (at 4 and 24 h after injection). Platelet endothelial cell adhesion molecule-1 (CD31) was stained with rhodamine (red) and nuclei were stained with DAPI (blue) for visualization. Dashed boxes are shown at 3X magnification at the bottom left of each image. Scale bar 20 µm. (**C**) Time-dependent effect of exogenous sEVs on gene expression in young mice. Gene expression was assessed 4 (blue bars and dots) and 24 h (red bars and dots) after treatment by PCR normalized to *Gapdh* and compared to the respective controls (black bars and dots) (CTR; set to 1.0). For display purposes, only one bar is presented. Results are shown as mean ± SEM. * p < 0.05 against CTR. One-way ANOVA with Dunnett’s multiple comparisons test.

Isolated sEVs from old mice were stained with PKH67 and injected into the tail veins of 3-month-old mice, and animals were sacrificed 0.5, 4, or 24 h later. Control animals were injected with 0.1% BSA in 0.9% NaCl. Brain sections were stained with anti-CD31 antibody and DAPI for localization of endothelial cells and cell nuclei, respectively, and observed under a fluorescence microscope (Figure 2A). PKH67-labeled sEVs (green fluorescence) were detected in brain tissue of mice treated with sEV but not in controls, confirming the specificity of the signal. Distribution of sEVs in brain tissue was not homogenous as observed by the agglomeration of green signals in cluster-like complexes. In general, the strongest signals were observed in circumventricular zones, the cortex, and the hippocampus. The staining was more abundant in anterior and medial regions, approximately between bregma 0.5 mm and −2.0 mm and gradually decreased towards the posterior part of the brain (Supplementary Figure S1). At 0.5 h, stained EVs usually colocalized with CD31^+^ vessels. At 4 and 24 h EVs were mainly localized out of the vessels and in many cases were also CD31^+^ (Figure 2B). 

### 3.3. sEVs from Old Animals Alter Gene Expression in Brain Tissue In Vivo

The expression of cell activation, senescence, and inflammatory genes was assessed in brain tissue of young mice injected with sEVs from old animals (sEV_Old_) for 4 and 24 h (Figure 2C). Compared to controls (0.1% BSA in 0.9% NaCl), a significant increase of genes related with glia activation (*Gfap* and *Cd68* but not *Iba1*) but not with brain inflammation (*Il-6*, *Il-1β*) was observed at 24 h post-injection. *Tnf-α* mRNA expression increased at 4 h but returned to control levels by 24 h, while *Tgf-β* mRNA increased at 24 h (Figure 2C). No significant changes were found in mRNA levels of the senescence marker *p16* or *iNOS* at the tested times (Figure 2C). GFAP, but not Iba1, protein levels were significantly induced at 24 h, as observed in a representative immunostaining (Supplementary Figure S2A,C). These changes were associated with morphological alteration of Iba1^+^ glial cells towards a reactive-like phenotype and were identified in the same brain regions where the sEVs localized (Supplementary Figure S2B). For the tissue-specific control, a panel of genes was investigated in liver tissue at 24 h, and only a significant decrease in *Il-6* was observed (Supplementary Figure S3). Based on these results, a 24 h treatment was selected for the expression study in young and aged mice after sEV delivery.

### 3.4. Differential Effect of Aged and Young sEVs on Gene Expression in the Brain

**Figure 3:**
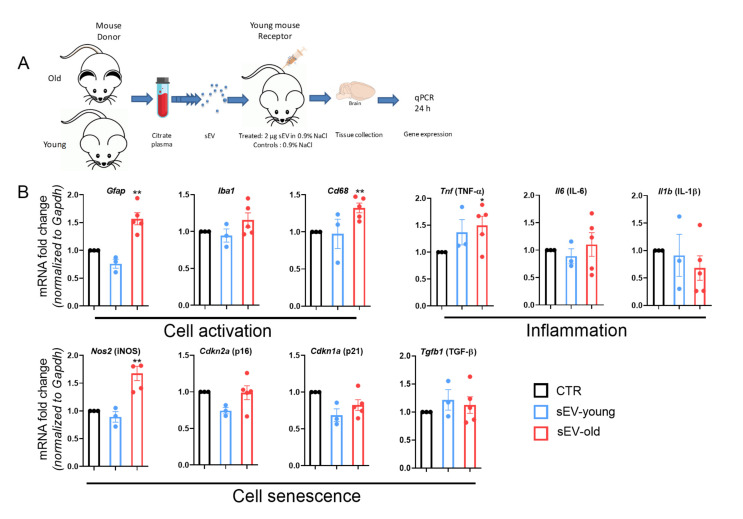
sEVs from old but not young mice induce brain glial activation in vivo. (**A**) sEVs were isolated from plasma of young or old donor mice. Two µg of unlabeled sEVs were injected into the tail veins of 3-month-old mice. Brains were collected 24 h after injection for gene expression assessment. (**B**) Gene expression of activation and senescence markers were analyzed by PCR and normalized to *Gapdh*. Results are shown as mean ± SEM. Single values are represented as dots. * p < 0.05, ** *p* < 0.01 against control (CTR). One-way ANOVA with Dunnett’s multiple comparisons test.

To evaluate whether aging of donor mice could induce a differential response in recipient mice, the expression patterns in the brains of young mice were assessed 24 h after injecting sEVs from young or old mice (Figure 3A). A significant increase in mRNA expression of cell activation markers, *Gfap* and *Cd68*, was observed after delivery of old but not young sEVs compared to controls (Figure 3B). mRNA levels of senescence markers, *p16* and *p21*, were unaltered but *iNOS* mRNA was upregulated 24 h after administration of sEVs from old animals (Figure 3B).

### 3.5. Uptake of Peripheral sEVs by Microglia and Astrocyte Cells In Vitro

**Figure 4:**
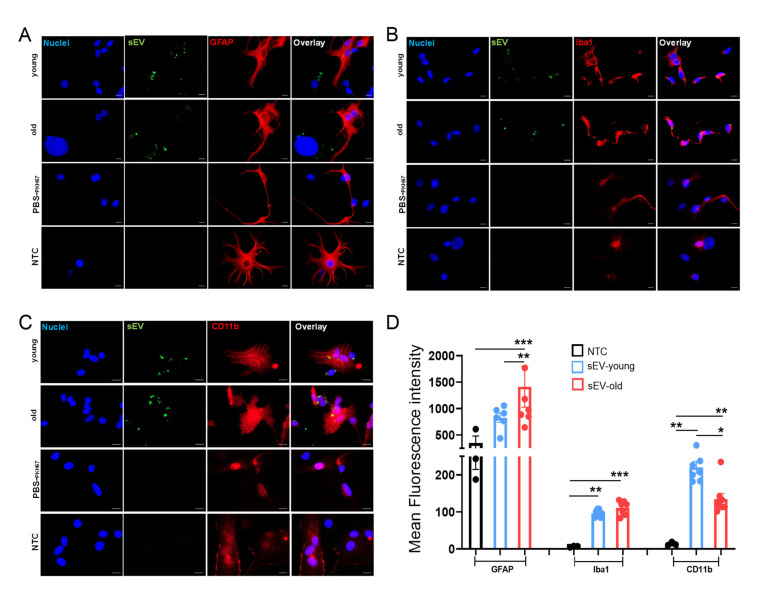
sEV-uptake and -mediated effects in vitro. Primary murine astroglia cells were incubated with PKH67-labeled sEVs (pseudo-green) from young (sEV_young_) and old mice (sEV_old_) for 24 h. Representative images of immunofluorescence staining for (**A**) GFAP (pseudo-red), (**B**) Iba1 (pseudo-red), and (**C**) CD11b (pseudo-red). Nuclei (blue) were counter-stained with DAPI Scale bar 50 µm. (**D**) Summary bar graphs of the relative mean fluorescence intensity (MFI) activation genes. NTC: Non-treated cells. Bars represent the mean ± SEM of MFI. * *p* < 0.05, ** *p* < 0.01, *** *p* < 0.001. One-way ANOVA with Dunn’s multiple comparisons test.

To determine whether a specific cell population of glia cells could be targeted by peripheral sEVs, an uptake study was designed using a primary mixed glial culture containing 80% astrocytes and 20% microglia treated with PKH-labeled sEVs. Astrocytes and microglia were immunostained with specific markers (GFAP, Iba1, and CD11b, respectively), nuclei were counterstained with DAPI, and colocalization with sEVs (green) was investigated by fluorescence microscopy. sEVs localized mostly in the cytoplasm compartment of recipient Iba1^+^ CD11b^+^ cells and in minor proportion by GFAP^+^ cells. However, sEVs were internalized by both populations, as observed by the presence of green dots and aggregations in both cell types (Figure 4A–C). Levels of Iba1 and CD11b increased greatly upon treatment with both sEV_old_ and sEV_young_, suggesting microglia as the primary target cells of exogenous sEVs. The effect on GFAP augmentation was observed only upon treatment with sEV_old_ (Figure 4D).

### 3.6. Effect of Aged and Young sEVs on Gene Expression In Vitro

**Figure 5:**
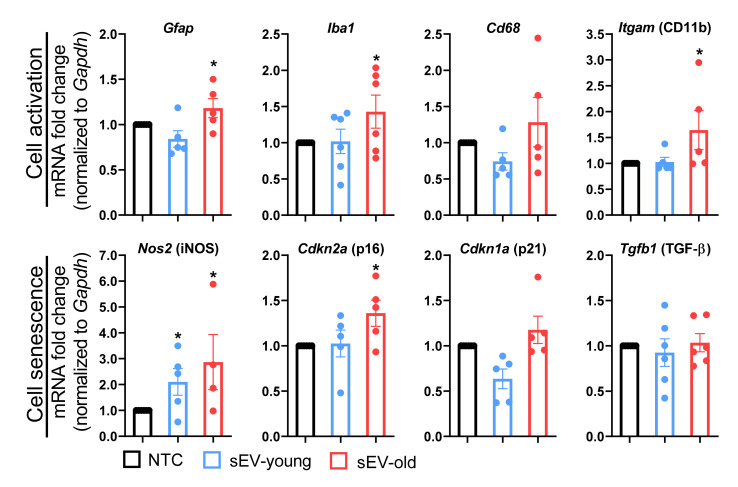
Differential effect of young and old sEV in vitro. Primary murine astroglia cells were incubated with sEVs from young (sEV_young_) and old mice (sEV_old_) for 24 h. Gene expression was assessed by PCR and normalized to *Gapdh*. Bars represent the mean ± SEM. NTC: non-treated cells. * *p* < 0.05. One-way ANOVA with Dunn’s multiple comparisons test.

To investigate longer-lasting effects, we used the in vitro model of primary-mixed cultures described before and investigated the expression of cell activation (*Gfap*, *Iba1*, *Cd11b*, *Cd68*) and senescence- (*iNOS*, *p16*, *p21*, *Tgf-β*) related genes after a 24 h treatment with sEVs from aged and young animals. Similar to the observations in vivo, treatment with sEV_old_ was more efficient than sEV_young_ in inducing expression of genes of the selected panel. Compared to non-treated cells, expression of *Gfap*, *Iba1*, *Cd11b*, and *p16* increased upon sEV_old_ delivery, while *iNOS* was induced by both sEV_old_ and sEV_young_ (Figure 5).

## 4. Discussion

Due to their ability to transfer proteins and genetic information horizontally [18], EVs are increasingly recognized as a mediator of blood-brain communication and intercellular communication in the CNS [19,20]. Accumulating evidence demonstrates involvement of sEVs in the regulation of inflammation and oxidative stress affecting a plethora of human pathologies [21]. For instance, misfolded proteins, disease-associated particles, such as α-synuclein, and prions can be transferred from origin to recipient cells via sEVs driving the progression of neurodegenerative disorders, including Alzheimer’s disease [22]. EVs are released by most of the CNS cells, including neurons, microglia, oligodendrocytes, astrocytes, and neural embryonic stem cells under healthy and pathological conditions [23,24]. Although the role of EVs in healthy aged brain remains largely unveiled, there is substantial circumstantial evidence linking EVs with the senescence-associated secretory phenotype and the immunological changes that contribute to driving aging [25,26]. However, the literature is heterogeneous regarding the effect of aging on secretion of EVs in humans. While a previous study reported that aging and cancer are associated with increased sEVs in serum [27], a cross-sectional and longitudinal study reported that circulating plasma EVs were decreasing with advancing age [7]. We found that the number of sEVs increased in the plasm of old compared to young mice and these sEVs harbor elevated markers of exosomal and endothelial origin, indicating that aging may alter the concentration, composition, and function of circulating EVs.

Further, EVs can cross the BBB by a variety of vesicular-mediated mechanisms, including specific transporters, adsorptive transcytosis, and a brain-to-blood efflux system [15]. Compared to larger EVs, sEVs are more likely to cross from periphery to brain, and therefore, they are considered as delivery vehicles of drugs into the CNS [28]. At the same time, this ability could result in negative effects when elevated levels of sEVs from the periphery access the parenchyma or when their content deviates from the normal [29]. For instance, peripheral-circulating inflammatory sEVs induce neuroinflammation associated with pro-inflammatory miRNAs, including miR-146a and miR-155, when reaching the cerebrospinal fluid [3,20].

Several invasive and non-invasive administration routes have been developed for the delivery of EVs and their content in the brain. All have proved to be useful for accessing the CNS, but the resulting biodistribution and clearance of EVs differ among methods, which makes some of them more suitable for therapeutic or experimental purposes [30]. In our hands, intravenous administration is the model that fits better with the analysis of peripheral-EV effects on CNS, because it allows the comparison with other reports such as the age-associated inflammatory changes, including glial cell activation, mediated by plasma of aged mice [31] and the effect on neuroinflammation caused by peripheral-circulating inflammatory exosomes delivered intravenously [3]. Our results demonstrate that intravenously injected sEVs localize to the brain of recipient mice, as demonstrated by the time-dependent accumulation of CD31^+^/PKH67^+^ sEVs in the brain parenchyma. Whether sEV accumulation of peripheral sEVs in the small capillary of the brain is due to a slower clearance in the brain compared to other organs, such as the liver, could not be determined with our experimental settings. It is known that clearance rates (t_1/2_) for sEVs from the blood are in the range of a few minutes [15]. Furthermore, sEVs are quickly removed from circulation by macrophages in the liver [32], which would support a lower clearance in the brain. The fact that accumulating vesicles in the brain express the endothelial marker, CD31, agrees with the inherent presence of CD31^+^ EVs in the plasma of aged mice as shown by WB analysis. Additionally, it may suggest that plasma EVs are actively transported to the parenchyma by the process of transcytosis, as already shown before [15].

Likewise, and in concordance with the localization results, a clear change in expression of genes related to glia activation (*Gfap*, *Iba1*, *Cd68*, *Tgf-β*) but not with inflammation (*Tnf-α*, *Il-6*, *Il-1β*) were observed in brain tissue. Immunostaining of representative brain slices confirmed upregulation of GFAP and increased amoeboid morphology of Iba1^+^ cells consistent with a reactive-like phenotype in the cortex region where EVs were localized. This strengthens the observations on glia cells as targets of peripheral-delivered EVs and their consequent activation, but a more comprehensive study is required to confirm these findings. Our results agree with a previous report showing microglia and astrocyte activation in mice after systemic delivery of serum sEVs from LPS-challenged donors [3]. In our hands, *Il-6* but not *Iba1* or *Cd68* expression was affected in the liver suggesting a tissue-specific response of macrophages to peripheral EVs. Other reports have demonstrated biodistribution of intravenously injected EVs in the liver, spleen, kidney, and lungs [33,34]; therefore, additional effects of aged sEVs in these organs could not be ruled out.

Changes in glial cell-mediated uptake and release of sEVs were recently reported to be involved in neurodegenerative diseases [35]. Here, we show that glial cells can be activated by interaction and internalization of peripherally injected sEVs. Most strikingly, a significant increase in *Gfap* transcription was observed after injection of sEVs from old but not young donor mice. Despite being consistent, treatment with stained and un-stained EVs displays differences in the degree of change in gene expression. This could be attributed to the already reported physical differences caused by EV membrane staining [36], which may alter their function. However, other factors such as interference of PKH67 in the EV quantification via colorimetric assays, or the interindividual differences in the control groups cannot be ruled out. These characteristics should be considered when comparing results among different publications.

Pioneer studies have developed the concept that aging and rejuvenation are transferable via blood constituents. In a landmark study from 2011, it was shown that connecting the blood vessel systems of a young and an old mouse caused the young animal to age significantly faster [37]. Furthermore, the same group showed that peripheral blood from young mice even reversed the impairment in cognitive function and synaptic plasticity of older mice [38]. The protocol used in this study for sEV enrichment excludes the possibility that the observed effects are due to other activation factors present in the circulation and support a role of sEVs in neuroinflammation, as observed during aging.

To further determine whether peripheral sEVs could be recognized by specific glial populations, we also used an in vitro model consisting of mixed primary astrocytes and microglia cells. Uptake analysis demonstrates that both populations interact with and take up sEVs within a short period of time, which then colocalize more often with microglia (Iba1^+^ CD11b^+^ cells). This may be explained by the phagocytic capacity of microglia cells, as highlighted by the high recognition and internalization of sEVs by Iba1^+^ populations in brain and spleen [39,40]. Alternatively, microglia were found to take up sEVs by a macropinocytotic mechanism, without induction of an associated inflammatory response [41], which also agrees with our current findings. In contrast to the strong changes observed under the in vivo settings, changes in gene expression observed with the in vitro approach were lesser. The discrepancy within the findings could be due to a necessary interaction between peripheral sEVs and the BBB, causing an indirect activation of glia cells, but also to an already activated state of microglia and astrocytes in the in vitro assay induced by the isolation procedure and culture conditions. In support of the first assumption, it is known that peripheral EVs can interact with the BBB, leading to changes in the barrier’s properties, and this could possibly alter the response of brain neural cells [42]. However, the increased *Tgf-β* expression in the primary mixed culture observed upon treatment with sEVs from old but not young donors suggests a specific effect of molecules on the surface and/or content of aged sEVs.

Altogether, our study indicates that peripheral-injected sEVs localize into brain tissue and induce activation of glial cells in the mouse. Furthermore, expression of *Gfap* and *Tgf-β* were significantly altered only after treatment with aged sEVs, suggesting differences in their surface and content mediated by aging of the donor mice. These alterations in sEV content might be involved in transferring of the aging phenotype via blood from old to young mice as shown in other studies, and might represent a putative target for the development of therapies aimed at reducing the onset and progression of aged-associated neuroinflammation and degenerative diseases.

## Figures and Tables

**Table 1:** Primer sequences used for quantitative RT-PCR SYBR Green protocol.

Primer	Forward	Reverse
*Cdkn2a* (p16)	*ctttgtgtaccgctgggaac*	*ctgaggccggatttagctct*
*Il1b* (IL-1β)	*gaagagcccatcctctgtga*	*ttcatctcggagcctgtagtg*
*Tnf* (TNF-α)	*gtctactgaacttcggggtgat*	*atgatctgagtgtgagggtctg*
*Cd68*	*ttctgctgtggaaatgcaag*	*gagaaacatggcccgaagt*
*Iba1*	*acagcaatgatgaggatctgc*	*ctctaggtgggtcttgggaac*
*Il10* (IL-10)	*atggtgtcctttcaattgctct*	*aggatctccctggtttctcttc*
*Tgfb* (TGF—β)	*tgcttcagctccacagagaa*	*tactgtgtgtccaggctcca*
*Il6* (IL-6)	*acaaagccagagtccttcagag*	*cattggaaattggggtagga*
*Nos2* (iNOS)	*tgactcccagcacaaagggctca*	*gcactctcttgcggaccatctcct*
*Gfap*	*agaaaggttgaatcgctgga*	*gccactgcctcgtattgagt*
*Gapdh*	*caacagcaactcccactcttc*	*Ggtccagggtttcttactcctt*

## Data Availability

Not applicable.

## Supplementary Materials

The following supporting information can be downloaded at: https://www.mdpi.com/article/10.3390/cells11040625/s1, Supplementary Figure S1. Images of coronal brain slices acquired using automatic tiling and stitching tools of the Axio Observer microscope (Zeiss). Supplementary Figure S2. Glial activation after sEV-old injection in vivo. Supplementary Figure S3. Panel of investigated genes in liver tissue at 24 h after sEV administration.
