# Supplementary material for: Small Extracellular Vesicles from Peripheral Blood of Aged Mice Pass the Blood-Brain Barrier and Induce Glial Cell Activation
Source: Cells. 2022 Feb 11;11(4):625. doi: 10.3390/cells11040625 (PMC8870085; doi:10.3390/cells11040625)

Supplementary Figure S1. Images of coronal brain slices acquired using automatic tiling and stitching tools of the Axio Observer microscope (Zeiss).

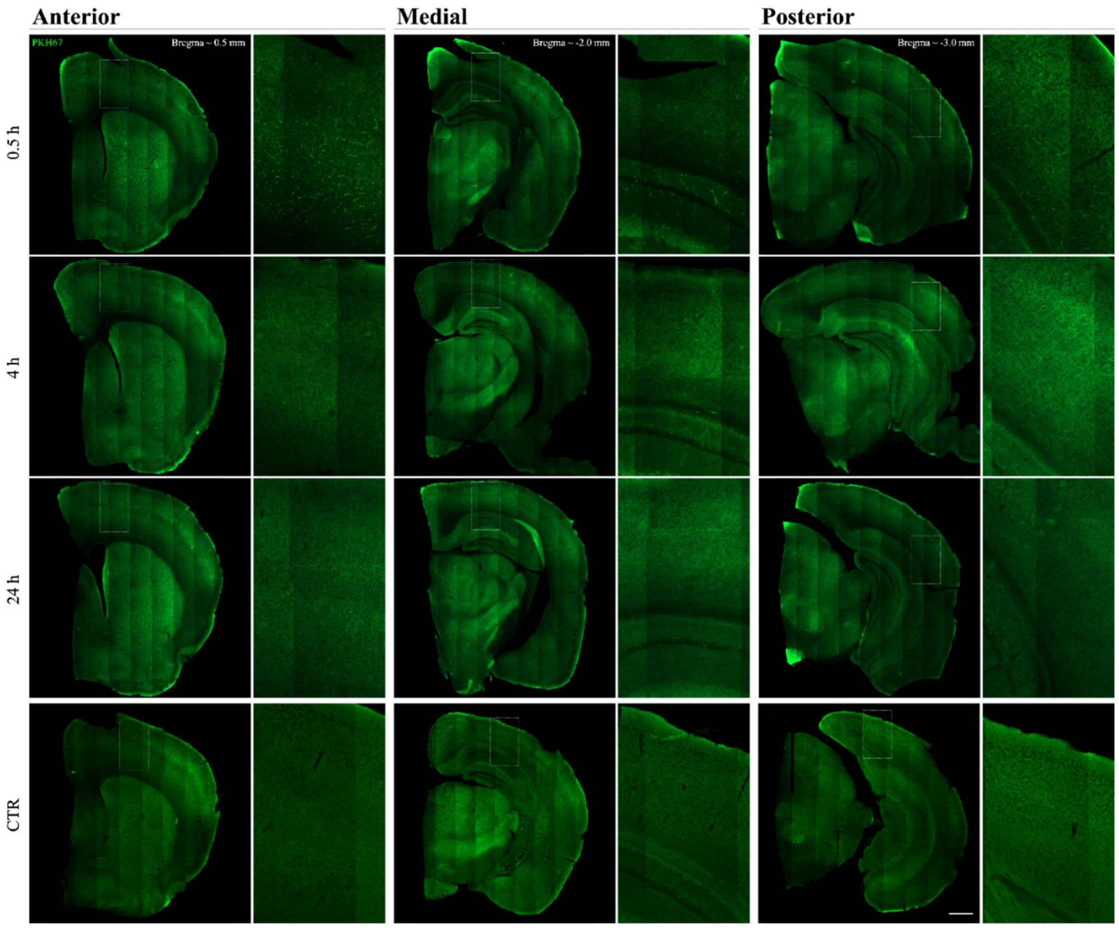

Supplementary Figure S2. Glial activation after sEV-old injection *in vivo*. **A)** Brains sections from non-treated (NTC) and sEV-old injected mice were immunostained following the protocol described in the methods section. Chicken anti-mouse GFAP antibody (pseudo green) (Encor, Cat-Nr. ABIN 4956156) and rabbit anti-mouse IBA1 antibody (pseudo red) (Wako, Cat-Nr. 016-20001) at a 1:500 dilution, and rhodamine donkey anti-chicken antibody and Alexafluor647 donkey anti-rabbit antibody at a 1:500 dilution were used. 1 μg/ml of DAPI was used for nuclei staining. Coronal sections in the medial brain (approximately in bregma -2.0 mm) of mouse cortex and hippocampus showing increased GFAP expression 24 h after injection of sEV-old compared to NTC. **B)** Magnification of the boxes in cortex and hippocampus showing amoeboid-like microglial somas (white arrowheads), and highly ramified morphology and thickening of astrocyte processes in the treated mouse compared to NTC. **C)** Summary of the GFAP and Iba1 fluorescence intensity from different areas of brain tissue (4 fields). Scale bar: A: 100 μm; B: 50 μm.

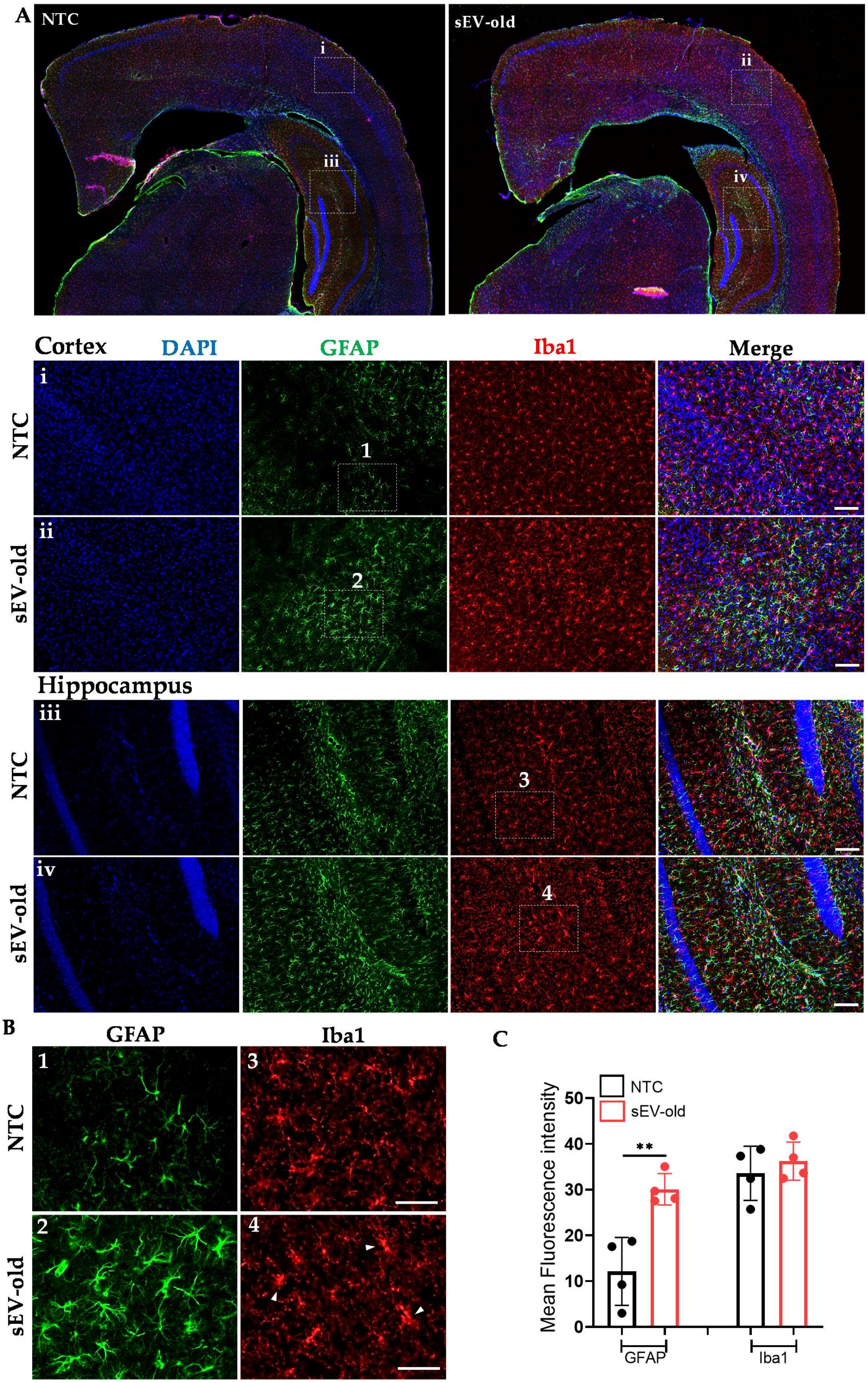
A
NTC
sEV-old
i
ii
iii
iv
Cortex
DAPI
GFAP
Iba1
Merge
NTC
sEV-old
1
2
Hippocampus
NTC
sEV-old
3
4
B
GFAP
Iba1
NTC
sEV-old
C
NTC
sEV-old
Mean Fluorescence intensity
0
10
20
30
40
50
**
GFAP
Iba1

Supplementary Figure S3. Panel of investigated genes in liver tissue at 24 h after sEV administration.

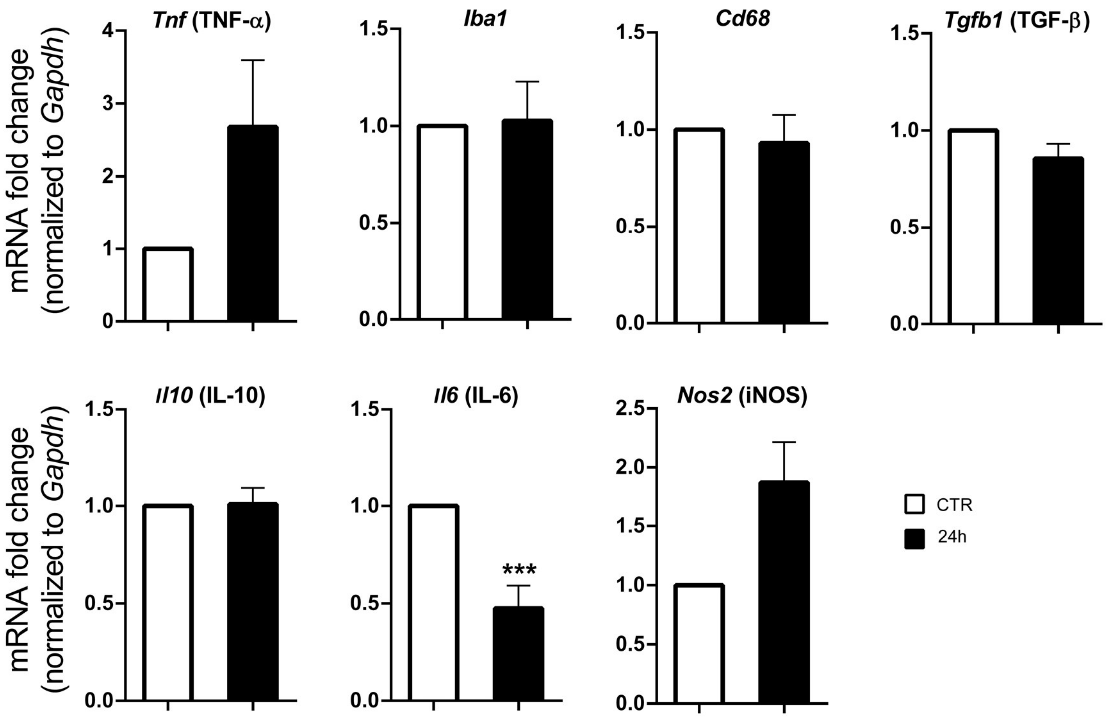

Supplement: Supplementary file 1 [file cells-11-00625-s001.zip › cells-1545085-supplementary.pdf]
